# A combination of virtual screening, molecular dynamics simulation, MM/PBSA, ADMET, and DFT calculations to identify a potential DPP4 inhibitor

**DOI:** 10.1038/s41598-024-58485-x

**Published:** 2024-04-02

**Authors:** Fateme Zare, Elaheh Ataollahi, Pegah Mardaneh, Amirhossein Sakhteman, Valiollah Keshavarz, Aida Solhjoo, Leila Emami

**Affiliations:** 1 Department of Medicinal Chemistry, School of Pharmacy, Shiraz University of Medical Sciences, Shiraz, Iran; 2 Medicinal and Natural Products Chemistry Research Center, Shiraz University of Medical Sciences, Shiraz, Iran; 3 Chair of Proteomics and Bioanalytics, Technical University of Munich (TUM), 85354 Freising, Germany; 4 Department of Quality Control of Drug Products, School of Pharmacy, Shiraz University of Medical Sciences, Shiraz, Iran; 5 Pharmaceutical Sciences Research Center, Shiraz University of Medical Sciences, Shiraz, Iran

**Keywords:** DPP4 inhibitor, Structure-based virtual screening, Molecular dynamics simulation, MM/PBSA, DFT, ADMET, Computational biology and bioinformatics, Drug discovery

## Abstract

DPP4 inhibitors can control glucose homeostasis by increasing the level of GLP-1 incretins hormone due to dipeptidase mimicking. Despite the potent effects of DPP4 inhibitors, these compounds cause unwanted toxicity attributable to their effect on other enzymes. As a result, it seems essential to find novel and DPP4 selective compounds. In this study, we introduce a potent and selective DPP4 inhibitor via structure-based virtual screening, molecular docking, molecular dynamics simulation, MM/PBSA calculations, DFT analysis, and ADMET profile. The screened compounds based on similarity with FDA-approved DPP4 inhibitors were docked towards the DPP4 enzyme. The compound with the highest docking score, ZINC000003015356, was selected. For further considerations, molecular docking studies were performed on selected ligands and FDA-approved drugs for DPP8 and DPP9 enzymes. Molecular dynamics simulation was run during 200 ns and the analysis of RMSD, RMSF, Rg, PCA, and hydrogen bonding were performed. The MD outputs showed stability of the ligand–protein complex compared to available drugs in the market. The total free binding energy obtained for the proposed DPP4 inhibitor was more negative than its co-crystal ligand (N7F). ZINC000003015356 confirmed the role of the five Lipinski rule and also, have low toxicity parameter according to properties. Finally, DFT calculations indicated that this compound is sufficiently soft.

## Introduction

Type 2 diabetes as non-insulin-dependent diabetes mellitus (NIDDM) is a progressive and complex disorder that hardly responds to treatment^1^. The decrease in insulin secretion due to the incomplete function of β-cells or the resistance of peripheral tissues (liver tissue, fat, and skeletal muscles) to insulin, is characteristic of type 2 diabetes which causes hyperglycemia disorder. The popular approach to treating type 2 diabetes is lifestyle modification and treatment with appropriate medications^2^. The World Health Organization (WHO) estimated that there will be about 300 million diabetic patients worldwide by 2025^3^. several classes of drugs are known for the treatment of diabetes such as Exenatide as glucagon-like peptide 1 (GLP 1) mimetic agents, Gliptins as dipeptidyl peptidase 4 (DPP4) inhibitors, Glitazones as peroxisome receptor antagonists. (PPAR-γ) agonists, Carbenoxolone as 11β-hydroxysteroid dehydrogenase-1 (11β HSD-1) inhibitor, Gliflozin as sodium/glucose co-transporter (SGLTs) inhibitors, etc.^4^. Among the relevant targets, DPP4 stands out as one of the enzymatic targets for type 2 diabetes. DPP4 is a serine protease that exists in both membrane-bound and plasma-soluble forms. This protease is a specific aminopeptidase for alanine and proline, which is responsible for the degradation of a series of biologically important peptides, including GLP-1 and GIP. Cleavage of GLP-1 increases glucagon release leading to a reduction of insulin secretion and increased blood glucose. DPP4 inhibitors can be effective compounds in controlling blood glucose levels by increasing GLP-1 levels^5^. Early DPP4 inhibitors were designed with relatively simple changes in proline structures^6^. By screening the existing compounds, many inhibitors with new structures were obtained such as Sitagliptin, Vildagliptin, Linagliptin, and Saxagliptin^7^. Since DPP4 inhibitors are GLP-1 mimicking rather than blocking DPP4, they disrupt the function of other dipeptide-degrading enzymes, so, probably cause undesired effects^8^ such as headache, upper respiratory tract infection, and nasopharyngitis. Pancreatitis and hypersensitivity are the main side effects of this category^9–11^. Sitagliptin and Vildagliptin exhibited genotoxicity effects since the antidiabetic drugs are long-term use drugs, this side effect should be considered^12^. Therefore, it is important to find new selective DPP4 inhibitors that bind tightly to the receptor and show lower side effects^13^. Recently, researchers applied computational methods to find new lead compounds by using virtual screening (VS) methods^14^. VS utilizes searching libraries of small molecules to identify potent structures by the ability to bind to a drug target (typically a protein receptor or enzyme). VS methods are divided into Ligand-based virtual screening (LBVS) and Structure-based virtual screening (SBVS)^15^ LBVS performs QSAR on a series of ligands obtained from laboratory research results while in the SBVS method, compounds screened from an interested database base and were docked on the protein target^16,17^. During decades much research was performed to find the DPP4 inhibitors, for example, Tanwar et al. introduced hydrazine analogs by using virtual screening workflow (VSW) and molecular dynamics simulation for DPP4 inhibitory activity selective against DPP8 and DPP9^18^. In addition, Hermansyah et al. found CH0002 as a potent DPP4 agent with low selectivity for DPP8 and DPP9 receptors using the VS in combination with the QSAR approach and artificial intelligence^19^. By the VS, the new non-peptides were found and evaluated as DPP4 inhibitors by Alonso et al.^20^.

In this paper, to identify a lead DPP4 inhibitor, SBVS in combination with molecular docking, molecular dynamics simulation (MDs), and molecular mechanics Poisson–Boltzmann surface area (MM/PBSA) approach was used. For the prediction of having adverse effects, molecular docking was performed on DPP8 and DPP9 in addition to the DPP4 enzyme. Furthermore, drug-likeness, physicochemical and pharmacokinetic properties, and Density functional theory (DFT) of the selected ligands were determined.

## Materials and methods

### Virtual screening and molecular docking

**Figure 1:**
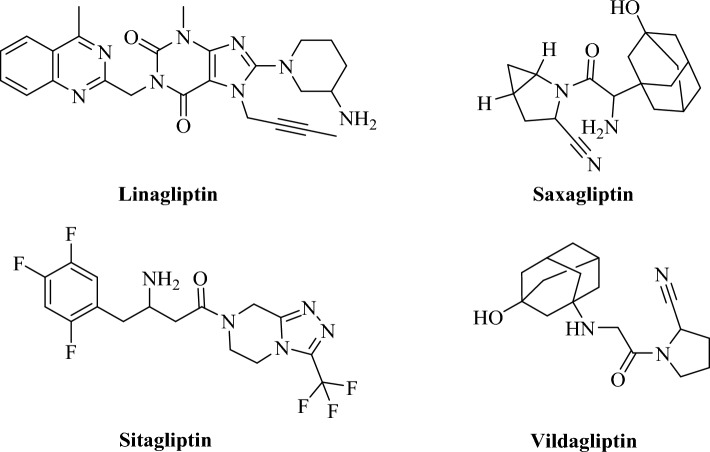
The FDA-approved DPP4 inhibitors.

FDA-approved DPP4 inhibitor drugs such as Sitagliptin, Vildagliptin, Saxagliptin, and Linagliptin were selected as templates for the beginning virtual screening (Fig. 1). Using the ZINC database (www.zinc.docking.org)^21^, the compounds were screened on ≥ 50% similarity^22^. A set of 200 compounds based on structural similarity was retrieved from the ZINC database including 1500000 compounds^23^. Molecules were converted to the pdbqt format to prepare for molecular docking study. The three-dimensional crystal structures of DPP4 (PDBID: 4a5s), N7F (co-crystal ligand of 4a5s), DPP8 (PDBID: 7a3k), QX8 (co-crystal ligand of 7a3k), and DPP9 (6eor), 9xh (co-crystal ligand of 6eor), were downloaded from the RCSB Protein Data Bank (https://www.rcsb.org/)^24^. The molecular docking study was run using Autodock Vina. Hydrogen atoms were added to the protein, and charges were assigned. The grid box size of 30 × 30 × 30 Å and exhaustiveness of 100 were set for screened ligands. The docking validation was accomplished by performing self-docking and the result was reported with root mean square deviation (RMSD) value^25^. The Discovery Studio Client 2016 was used to visualize the interactions and binding poses of the compounds.

### Molecular dynamics simulation

The simulations were conducted using GROMACSv2021 (http://www.mdtutorials.com/gmx/complex/index.html) applying an AMBER force field^26^. sufficient water molecules were replaced by counter ions. Periodic boundary conditions (PBC) were applied to all three directions of the system. Initially, the steepest descent algorithm was used for energy minimization^27^. Then, the system was equilibrated by NVT and NPT ensembles respectively. The equilibration at constant volume (NVT) was done with a coupling constant of 0.1 ps for 100 ps at a steady temperature of 300 K recruiting the Berendsen thermostat algorithm. The equilibration at constant pressure (NPT) was subsequently carried out with a coupling constant of 5.0 ps for 1 ns in the pressure of 1 bar maintained by the Parrinello–Rahman barostat. Particle Mesh Ewald (PME) was utilized to calculate the range of the interactions. Long-range electrostatic interactions and short-range non-bonded interactions were calculated within a 12 Å cut-off. Two simulations were performed with durations of 200 ns. The analysis of Root Mean Square Deviation (RMSD), Root Mean Square Fluctuation (RMSF), the Radius of gyration (Rg), the number of hydrogen bonds, and principle component analysis (PCA) were analyzed through the MD trajectories^28^.

### MM-PBSA calculation

The combination of molecular dynamics simulations and thermodynamic techniques such as Mechanic/Poisson-Boltzmann Surface Area (MM-PBSA) led to the measurement of the total binding free energy between ligand and protein as follows^29^:

$$\Delta {\text{G}}_{{{\text{binding}}}} = \, \Delta {\text{G}}_{{{\text{MM}}}} + \, \Delta {\text{G}}_{{{\text{sol}}}} {-}{\text{ T}}\Delta {\text{S}}$$

where, G (complex) is the total free energy of protein–ligand complex and G (receptor) and G (ligand) are the free energies of the isolated protein and ligand in a solvent, respectively. The total free energy for each of the three mentioned entities (complex receptor or ligand) could be calculated from its molecular mechanics’ potential energy (∆G_MM_) plus the energy of solvation (∆G_sol_)^30^. The intermolecular van der Waals (ΔE_vdW_), electrostatic interactions (ΔE_elec_), and nonpolar solvation energy (ΔE_np_) are favorable for binding. Still, polar solvation-free energy (ΔE_pol_) and the configurational entropy (− TΔS) are unfavorable in ligand–protein binding. Thus, the MM/PBSA GROMACS was used to calculate the total free binding energy of the ligand–protein complex through the MD trajectories^31^.

### ADMET profile

In-silico investigation including physicochemical properties and pharmacokinetic properties such as absorption, distribution, metabolism, excretion, and toxicity for selected DPP4 ligands, its related co-crystal, and some FDA-approved drug ligands, was accomplished. The results were obtained from SwissADME and the preADMET online servers (http://preadmet.bmdrc.org/).

### DFT studies

Gaussian 09 was used to analyze the Density Functional Theory (DFT) of selected DPP4 inhibitor candidates used and visualized through Gauss view 6.0. The optimization of the structural coordinates of the selected compounds was performed by using the B3LYP/6–31 G (d,p) level basis set without any symmetrical constraints. The electrostatic surface potential, the HOMO–LUMO energy, and thermochemical parameters (thermal Energies, thermal Enthalpies, thermal Free Energies, hardness, softness, Ionization energy, and electron affinity) of the ZINC000003015356 were obtained from the optimized geometry.

## Results and discussion

### Virtual screening and molecular docking studies

**Figure 2:**
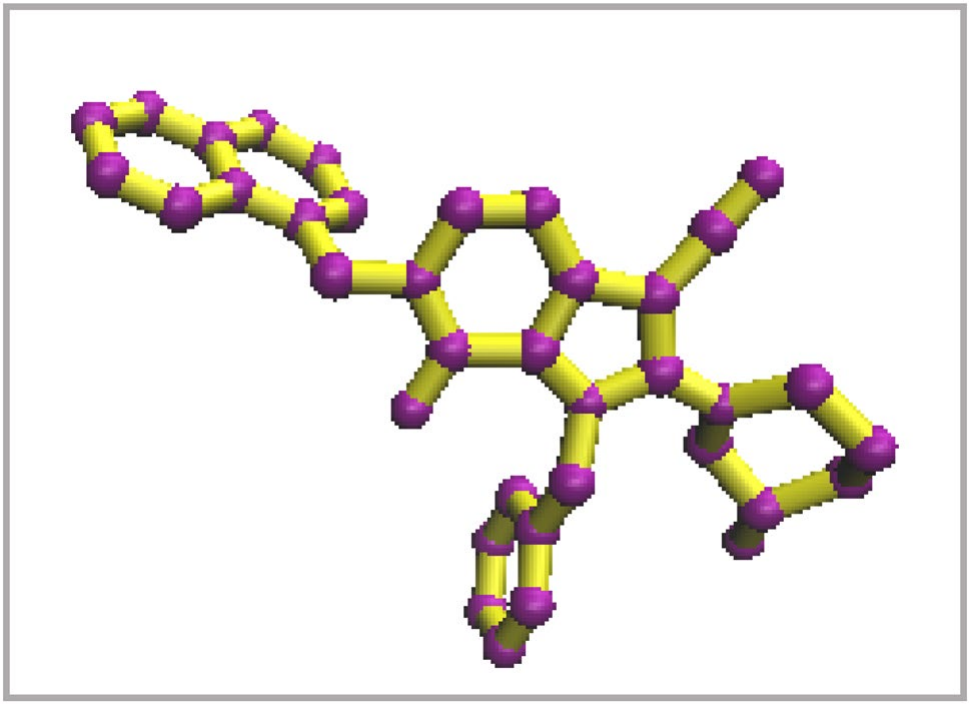
The superimposition of co-crystal and docked ligand in the active site 4a5s.

Molecular docking was conducted to characterize the molecular interactions and binding affinity of the protein–ligand complex^16^. The top-ranked ligands were grouped and filtered based on the affinity and appropriate orientation within the active site of the protein. Thereby screening the large library of ZINC database containing millions of known compounds, 200 compounds with a similarity of ≥ 50% were screened and molecular docking was performed to investigate their affinity to the DPP4 enzyme. The accuracy of molecular docking was confirmed by the re-docking simulation of the co-crystal ligand (N7F) in the active site of the DPP4 (4a5s) enzyme^30^. The root means square deviation (RMSD) value was obtained at 0.4 Å. The superimposition of the co-crystal ligand and docked N7F in the active site of 4a5s is shown in Fig. 2. The native ligand (N7F) was redocked back to the active site of the 4a5s enzyme to evaluate the docking protocol. Figure 2 shows the excellent superimposition of co-crystal ligand and docked ligand and it can validate the docking procedure. The RMSD of docking for N7F compared to its coordination in the crystal structure was very low value (0.31) and showed the reliability of the docking procedure The RMSD of docking for N7F compared to its coordination in the crystal structure was very low value (0.31) and showed the reliability of the docking procedure.

**Figure 3:**
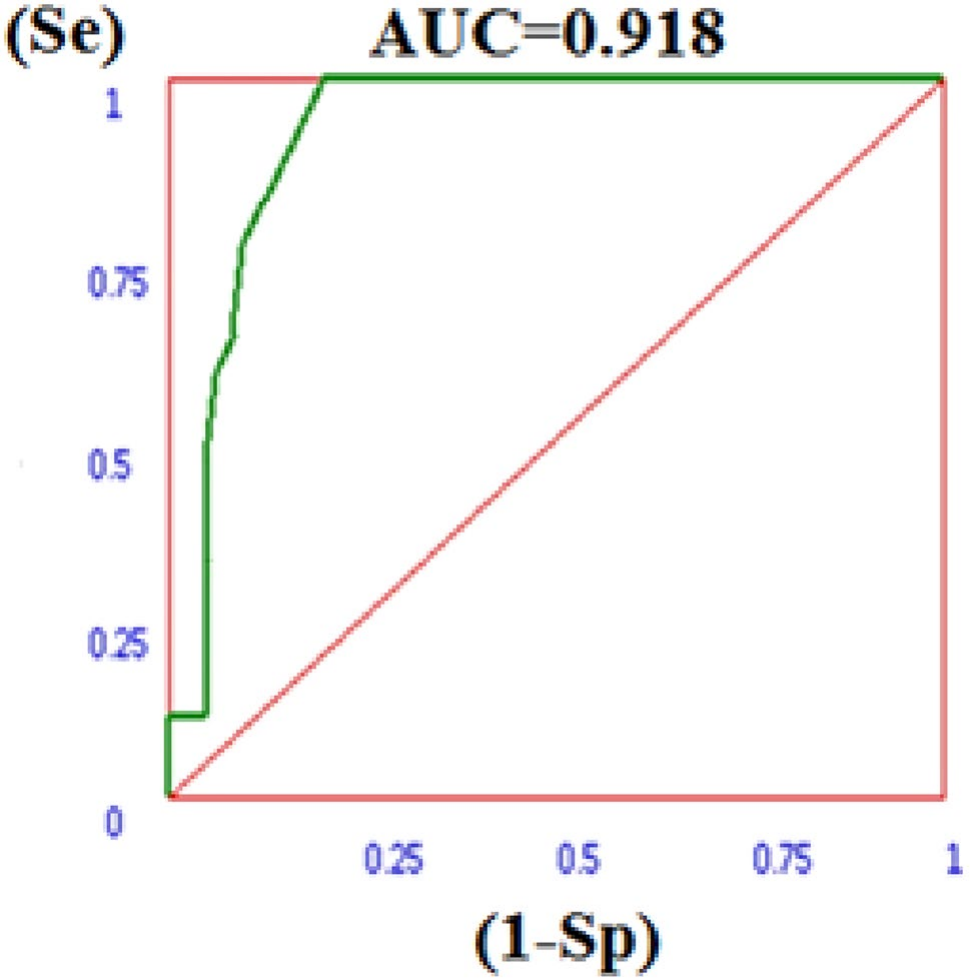
ROC curve for DPP4 (4a5s) receptor. (Se): Sensivity, (1 − Sp): (1 − specificity).

The Receiver Operating Characteristic (ROC) diagram is a graph to validate the ability of docking to discriminate between active and decoy compounds. ROC curve shows the true positive rate (sensitivity) vs false positive rate (1-specificity). The result of ROC is depicted in Fig. 3, AUC of 0.918 was obtained based on binding affinity scores and shows the success of the docking protocol implemented in distinguishing actives from inactive structures.

**Figure 4:**
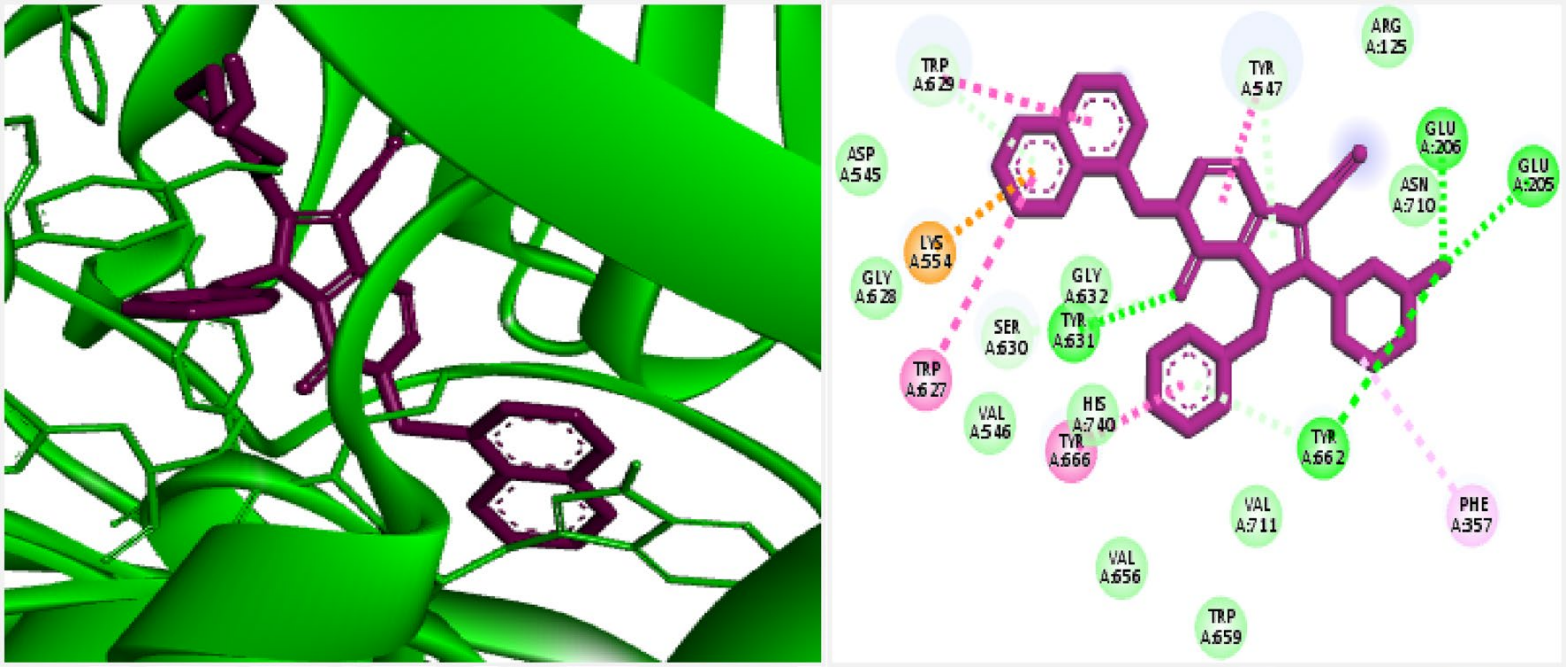
Interaction and orientation of co-crystal ligand in the binding pocket of DPP4 (pdb:4a5s).

The 3D, and 2D structures and interactions of internal ligands of DPP4 (N7F) are shown in Fig. 4. Based on the results, the binding energy for N7F was obtained at − 10.5 kcal mol^−1^ and established the tight bindings in the active site of 4a5s through conventional hydrogen bonding and π–π interactions. The key residues in the binding site of DPP4 are Lys554, Trp627, Trp629, Tyr547, Tyr666, Tyr 631, Glu206, Glu205, Tyr662.

**Figure 5:**
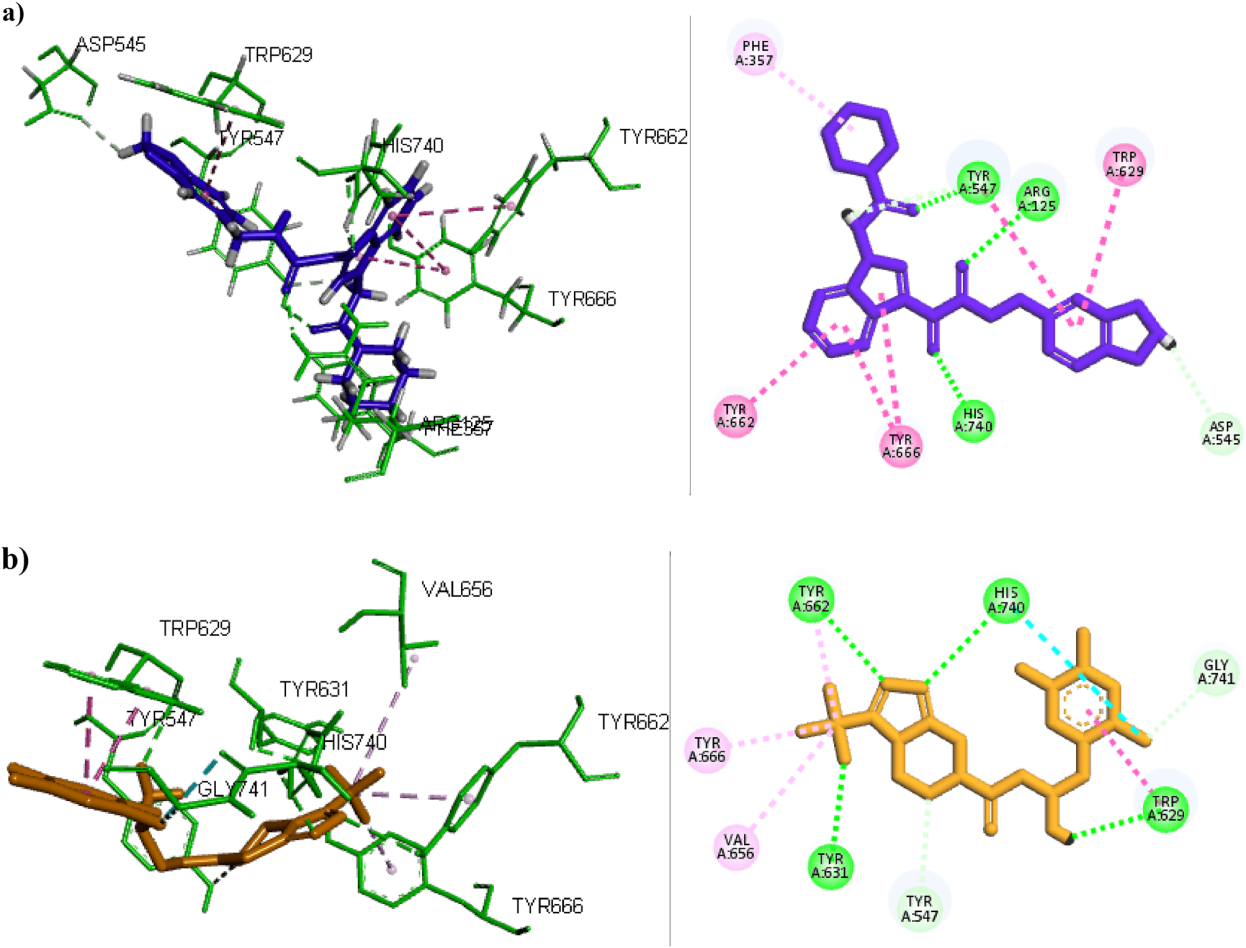
Interaction and orientation of (**a**) selected ligand ZINC000003015356, (**b**) Sitagliptin in the active site of DPP4.

Among the 200 docked compounds, 30 compounds showed binding energy in the range of − 8 to − 10.8 kcal mol^−1^, and amongst, the compounds with the highest binding energy (> − 10.0 kcal mol^−1^) were considered. The docking results of top-ranked compounds and Sitagliptin in the active site of 4a5s are shown in Table 1. As can be observed the binding energies for ZINC000003015356, ZINC000002876364, and ZINC000003007727 are − 10.8, − 10.0, and − 10.1 kcal mol^−1^. It can be understood that the binding energy of ZINC000003015356 was higher than other selected compounds, N7F and Sitagliptin as reference drugs. To select the lead compound, the interactions and key residues of ligands in the active site of 4a5s were considered and presented in Tables 1 and 2. The compound ZINC000003007727 formed a hydrogen bond with the residues of Arg125 and His740 and π–π stacking interactions with Tyr662, Tyr 666, and Trp629 residues. Also, ZINC000002876364 formed a hydrogen bond with His740 and Ser630 and participated in the π-stacking interactions with Tyr662, Tyr666, and Trp629 residues. The analysis of docking results for ZINC000003015356 displayed that this compound made the hydrogen bond interaction with the residues of His740, Arg125, and Tr547 and π–π stacking with Tyr662, Tyr666, Tyr547, Trp629 residues. The involved key residues in the active site of the enzyme for ZINC000003015356, namely Tyr662, Tyr666, Tyr547, and Trp629 were similar to N7F. Consequently, the ligand ZINC000003015356 with the highest docking score and appropriate interaction in the active site of DPP4 was selected as the proposed DPP4 inhibitor. The interactions and binding poses of ZINC000003015356 are given in Fig. 5a. For further analysis and comparison of the selected combination with drugs available in the market, docking of Sitagliptin was also done, and the results are shown in Fig. 5b. Sitagliptin interacted with Tyr662, Tyr 632, Trp 629, and His 740 as hydrogen bonding and His 740 and Trp 629 as π interactions. Analysis of docking outputs for Sitagliptin showed that the key residues and their surrounding residues were similar to ZINC000003015356, which confirmed the suitable selection of lead compound.

**Table 1:** The Docking score of some of the docked compounds in the active site of DPP4 (pdb:4a5s).

Compounds	Structure	Residues involved in hydrogen bonding	Distance (Å)	Docking score (kcal mol^−1^)
ZINC000003015356		Arg125, Asp545, Tyr547, His740	2.91, 3.52, 3.70,3.77	-10.8
ZINC000002876364		Arg125, Asp545, Trp629, Ser630, His740	2.88, 3.22, 3.70, 3.69,3.65	-10
ZINC000003007727		Val546, Ser630, His740	3.3, 3.16, 3.70	-10.1
N7F		Glu206, Glu205, Tyr662, Tyr663	2.56, 2.66, 2.34, 2.25	-10.5
Sitagliptin		Tyr662, His740, Tyr631, Trp629	2.83, 3.02, 3.69, 3.56	-9.2

**Table 2:** The hydrophobic π–π and hydrogen bond interactions for top-ranked compounds in the active site of DPP4 (pdb:4a5s).

Compounds	Residues involved in π–π and hydrophobic interaction	Ligand involved moiety	Type of interaction
ZINC000003015356	Arg125, Asp545, Tyr547, His740	Carbonyl groups	Hydrogen bond
	Tyr547, Trp629, Tyr662, Tyr666	Indoline and phenyl rings	π–π stacked, π–π T-shaped
	Phe357	Piperidine ring	π–Alkyl
	Glu206, Val546, Lys554, Gly628, Ser630, Tyr631, Gly632, Val656, Trp659, Arg669, Asn710, Val711	–	Hydrophobic interactions
ZINC000002876364	Arg125, Asp545, Trp629, Ser630, His740	Carbonyl groups	Hydrogen bond
	Trp629, Tyr662, Tyr666	Phenyl ring	π–π stacked, π–π T-shaped
	Tyr547, Trp627, Tyr666	Morpholine and Piperidine rings	π–Alkyl
	Val546, Lys554, Gly628, Tyr631, Gly632, Val656, Trp659, Asn710, Val711, Gly741	–	Hydrophobic interactions
ZINC000003007727	Val546, Ser630, His740	Carbonyl group	Hydrogen bond
	Trp629, Tyr662, Tyr666	Phenyl ring	π–π stacked, π–π T-shaped
	Tyr547, Tyr627, Tyr666	Morpholine and Piperidine rings	π–Alkyl
	Arg125, Asp545, Lys554, Gly628, Gly632, Tyr631, Val656, Trp659, Val711	–	Hydrophobic interactions
N7F	Lys554, Trp627, Tyr666, Trp629, Tyr547	–	π–cation, π–π stacked
Sitagliptin	Trp629, His740, Tyr666,Val656, Tyr662	Trifluoromethyl, triflourophenyl ring	π–π stacked, Halogen bonding, π–Alkyl

**Figure 6:**
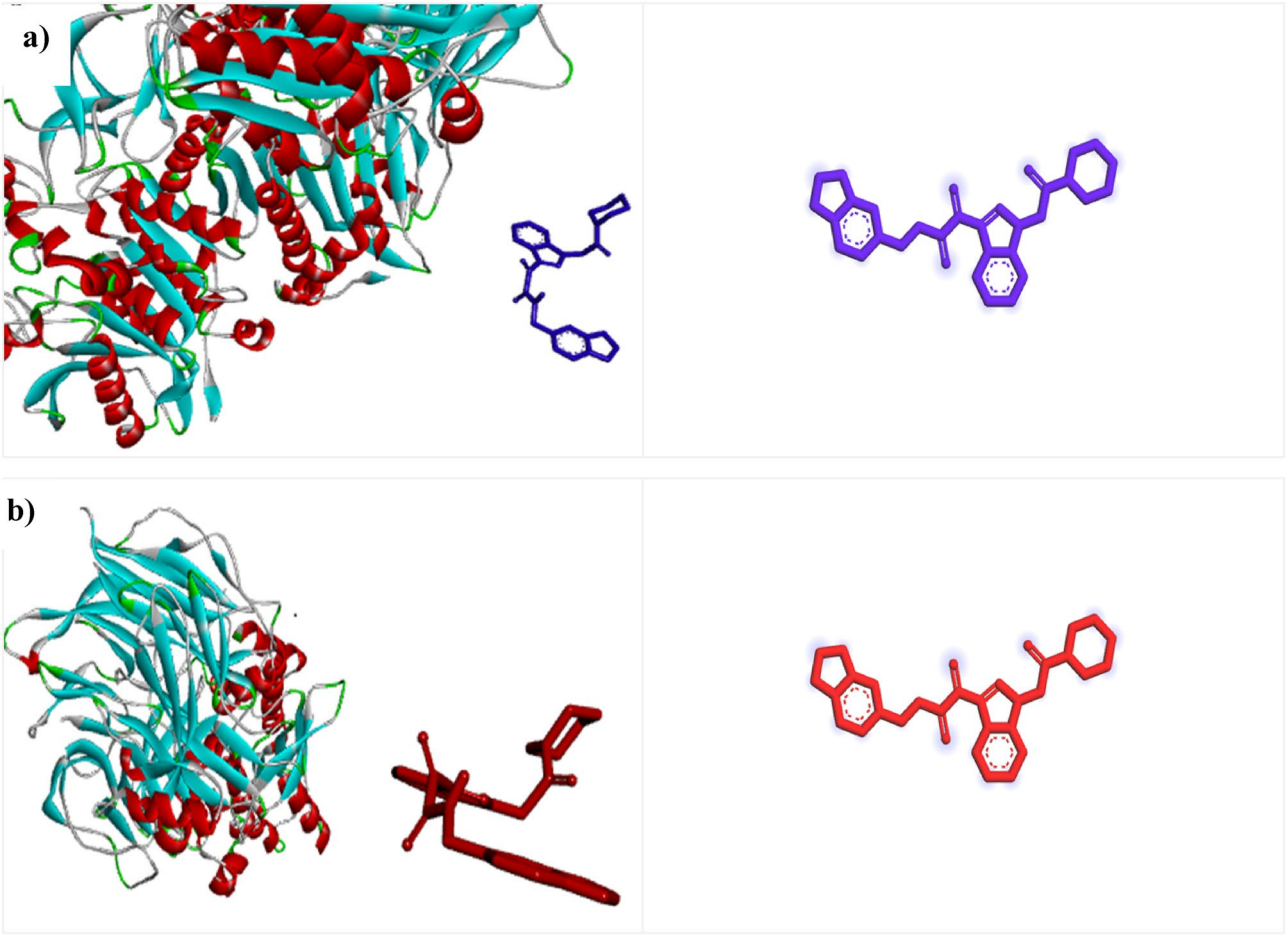
Consideration of interaction of selected DPP4 ligand in the active site of (**a**) DPP8 (PDBID: 7a3k) and (**b**) DPP9 (PDBID: 6eor).

As previously mentioned, one of the assumptions for the side effects of DPP4 inhibitors is interference with DPP8 and DPP9 enzymes. Therefore, docking studies of selective ligand, ZINC000003015356, were performed on DPP8 (PDBID = 7a3k) and DPP9 (PDBID = 6eor) receptors. The binding energies for ZINC000003015356 on DPP8 and DPP9 were obtained zero. As can be seen in Fig. 6, the proposed ligand does not interact with any of the DPP8 and DPP9 and is not placed in the binding pocket of these receptors.

**Figure 7:**
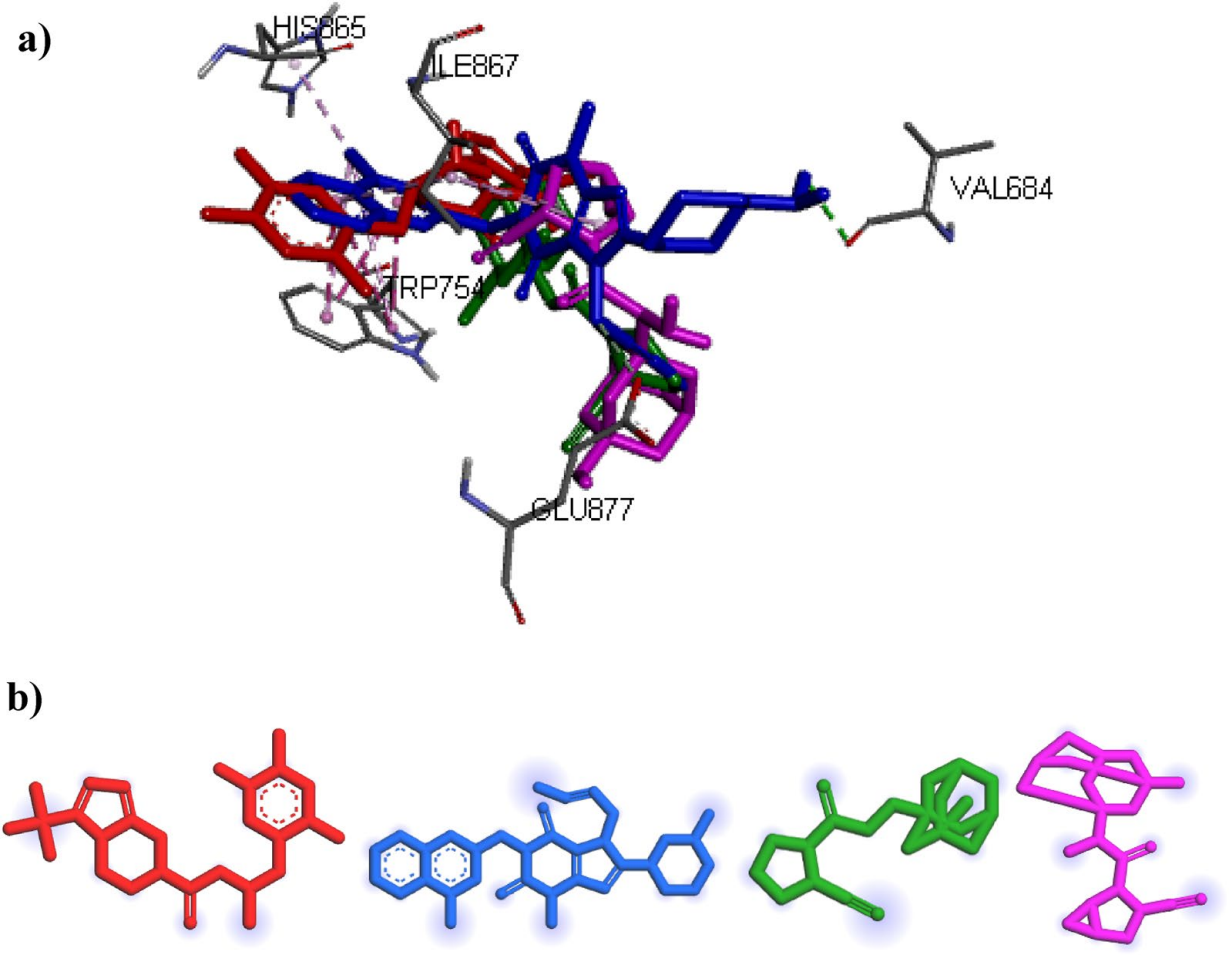
The 3-D interactions of FDA-approved in the active site of (**a**) DPP8 (7a3k) and (**b**) DPP9 (6eor): Sitagliptin (Red), Linagliptin (blue), Vildagliptin (green), saxagliptin (pink).

In contrast, the molecular docking of FDA-approved compounds was done on DPP8 and DPP9 enzymes. the docking results of these compounds on DPP8 enzyme showed that all compounds are placed in the active site of 7a3k. The binding energy for Sitagliptin, Linagliptin, Vildagliptin, Saxagliptin were obtained: − 9.8, − 9.3, − 8.1, − 7.7 kcal mol^−1^, respectively. Also, the results showed that all the compounds are not placed in the binding site of DPP9 and have no interaction with it, and their binding energy was obtained zero. The 3-D interactions of FDA-approved compounds on DPP8 (7a3k) and DPP9 (6eor) are shown in Fig. 7. It was already mentioned that one of the hypotheses of side effects related to current DPP4 drugs is their interaction with other receptors. The results of molecular docking confirmed that these compounds affect the DPP8 receptor, while the selected ligand (ZINC000003015356) did not affect receptors DPP8 and DPP9. This event can be a promising point for ZINC000003015356 as an appropriate lead DPP4 inhibitor.

Taken to gather, one promising compound (ZINC000003015356) established strong hydrogen bonds and hydrophobic interactions with important residues, Arg125, Asp545, Tyr547, His740, Glu206, and Ser 630 at the binding site of the DPP4 enzyme which is essential for selectivity^19,32^. ZINC000003015356 had better docking scores than that of the FDA-approved drugs for diabetes, Sitagliptin. Molecular docking results on DPP8 and DPP9 showed low binding energy and interaction with these enzymes. The ZINC000003015356 hit compound based on visualization results deserves to be as the best DPP4 hit compound.

### Molecular dynamic simulation results

**Figure 8:**
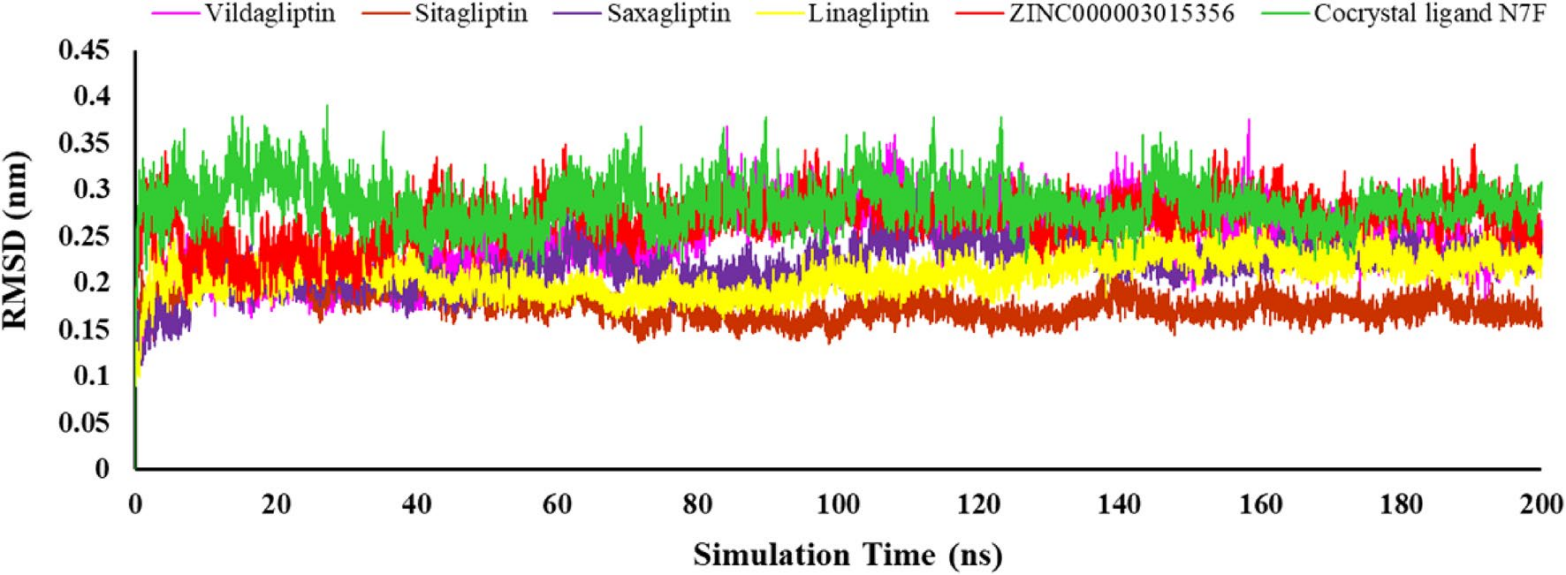
RMSD backbone for protein complexes of different ligands within DPP4 inhibitors.

The molecular dynamic simulation was used to investigate the dynamic behavior of the studied systems to confirm the results of the molecular docking^33^. The MD study was run on the top-ranked docked DPP4 ligand, namely ZINC000003015356. The RMSD, RMSF, Rg, and number of hydrogen bonding are given in Fig. 8. The RMSD calculation estimated the conformational fluctuations of the protein–ligand backbone atoms and stability of the simulated system during 200 ns of simulation time^34^. According to Fig. 8, after initial slight fluctuations due to kinetic shock, all systems reached to steady state. Almost all systems considered in molecular dynamics studies undergo an initial kinetic shock. The RMSD plot results showed that the chosen ligands in all systems reached a stable level, suggesting that the ligands exhibited consistent stability within the active site of the protein. Therefore, it can be concluded that the ligand–protein complex causes no significant conformational changes in the protein structure.

**Figure 9:**
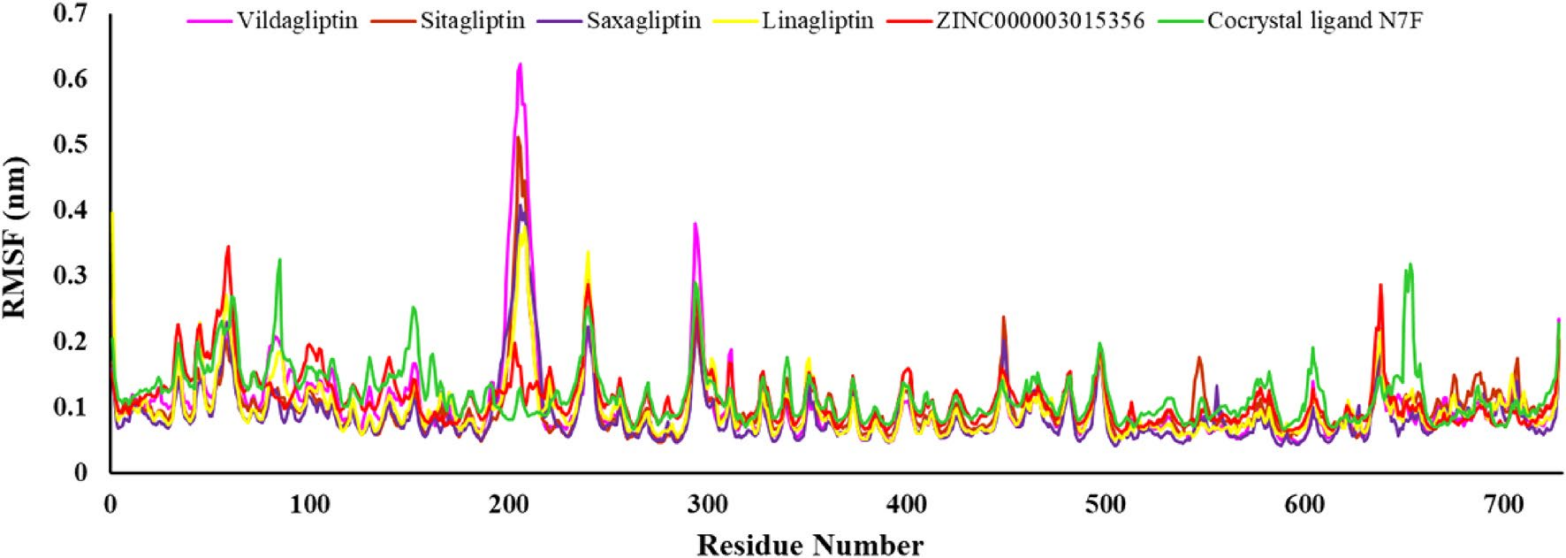
The RMSF plot of the complexes between positive references, cocrystal ligand, and ZINC000003015356 with DPP4 receptor.

The RMSF analysis determined the fluctuations of amino acid residues with the calculation of the average of their atoms^35^. Small RMSF values indicated less conformational changes of the protein in the complex with the ligand. The RMSF results of the complexes between positive references, cocrystal ligand (N7F), and ZINC000003015356 with DPP4 receptor are shown in Fig. 9. Six complexes displayed similar patterns and none of the amino acid residues had an RMSF greater than 0.4 nm in the active site of receptor. It was expected that the loop, C-terminal, and N-terminal regions showed more fluctuations compared to the residues in the binding site and were involved in ligand–protein interactions. The RMSF analysis for ZINC000003015356 showed that key amino acids namely Tyr662, Tyr666, Tyr547, and Trp629 had small RMSF values. The RMSF plots for the unbounded and bounded protein confirmed binding of the ligand to the protein did not change the protein structure^36^. Furthermore, the residual fluctuations less than 0.4 nm, indicated that the ligand–protein complex had no significant effect on the protein backbone, which was consistent with the RMSD results.

The majority of peaks were identified for Vildagliptin, Saxagliptin, Sitagliptin, and Linagliptin in residues 180–210, which are not associated with the enzyme's active site. These findings suggest that essential interaction between the ligand and binding pocket may contribute to preserving the protein's stability. The ZINC000003015356 ligand compareed to positive references (Vildagliptin, Saxagliptin, Sitagliptin, and Linagliptin) shows no significant changes in RMSF value.

**Figure 10:**
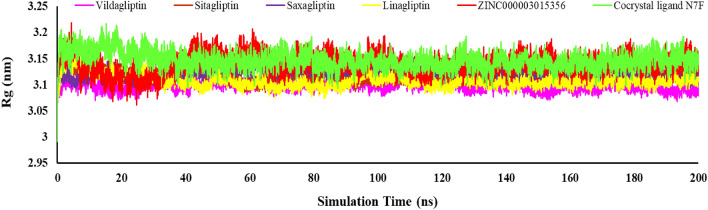
The Rg for DPP4 receptor in complex with positive references, cocrystal ligand, and ZINC000003015356 compounds during the simulation time.

The level of protein size change or its compactness in binding to the ligand during the MD simulation run was calculated with the Rg parameter^37^. The lower values of Rg indicated the higher compactness of the protein–ligand complex and the stability of the system. As shown in Fig. 10, Rg changes for ZINC000003015356 was in the same range of positive references. Vildagliptin and Linagliptin showed lower Rg in the range of 3.09 and 3.10 nm.

**Figure 11:**
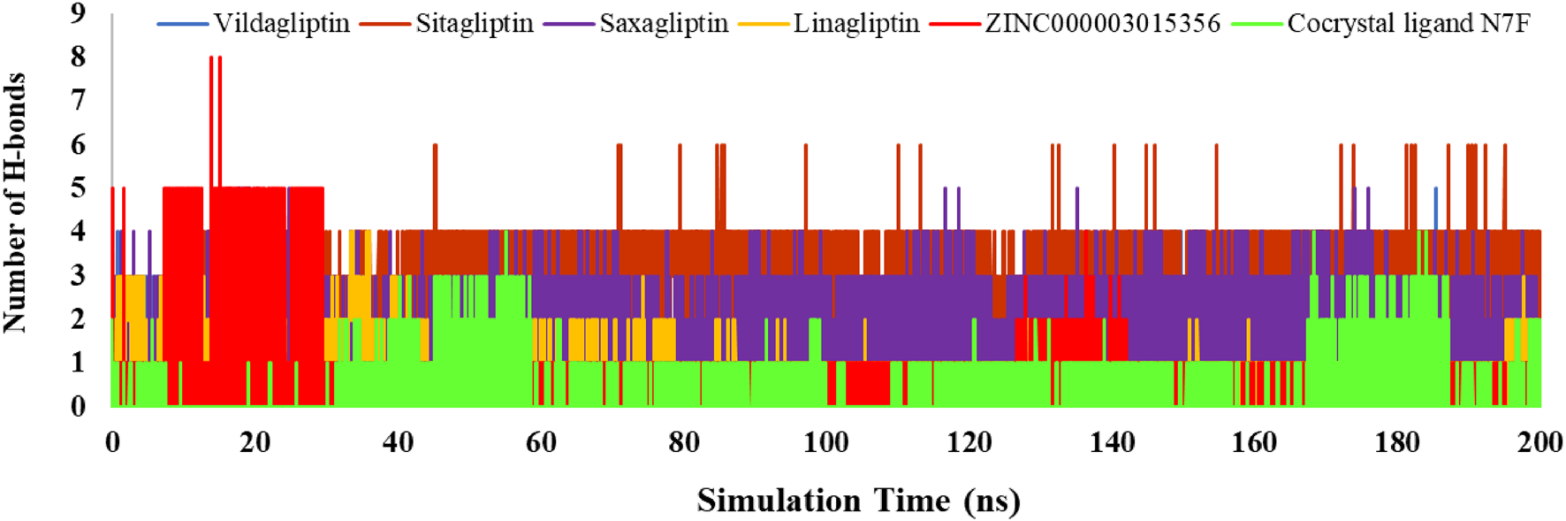
The total number of hydrogen bonds of ZINC000003015356, co-crystal ligand N7F, and positive references.

The examination of the total hydrogen bond interaction between each ligand with 4a5s during the simulation was also computed in Fig. 11. According to the results, the range of H-bonds for ZINC000003015356 was between 0 and 8, and for N7F was between 0 and 4 in the simulation time. The positive references showed good H-bond interaction in the active site of the protein. Therefore, the selected ligand formed a higher number of hydrogen bonds compared to the internal ligand through simulation time. In general, the analysis results of the MD simulation agreed with the docking study results.

**Figure 12:**
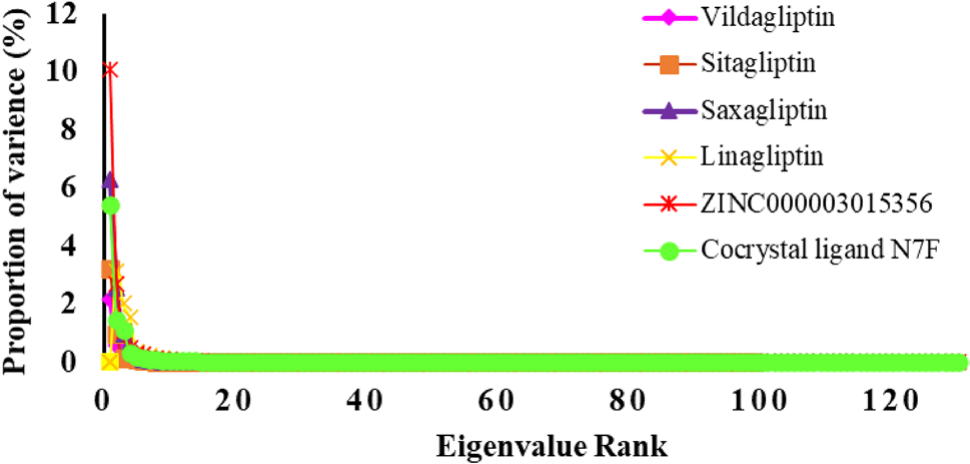
plot of the eigenvalue rank of all complexes.

**Figure 13:**
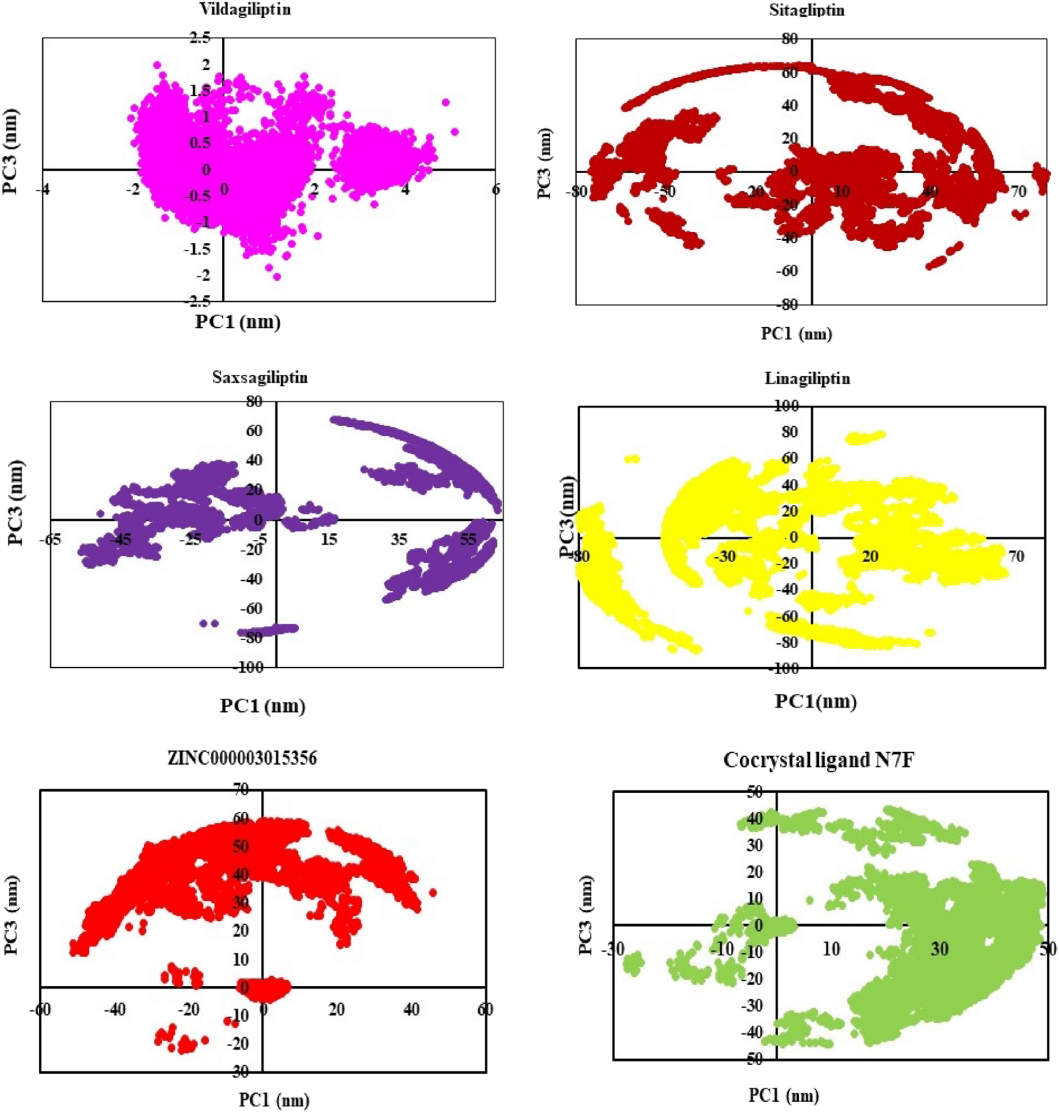
The scree plot for PC1 versus PC3.

Principal component analysis (PCA) or essential dynamics was one of the advanced methods in MD simulations^38^. For instance, biological macromolecules with numerous degrees of freedom, like proteins, may undergo significant conformational changes to adopt complex shapes and demonstrate a wide range of activities^39^. The examination of significant coordinated movements occurring during ligand binding is facilitated by the PCA analysis depicted in Figs. 12 and 13. In this study, the eigenvectors were acquired via performing diagonalization upon the matrix. Figure 12 shows the eigenvalues produced from the diagonalization of the covariance matrix of atomic fluctuations against the appropriate eigenvector in decreasing order. Based on Principal Component Analysis (PCA) findings, it can confidently be concluded that the six investigated complexes exhibit less motion and maintain a stable interaction. Another method for determining the dynamics of protein–ligand interactions is to create 2D projection plots using PCA. Figure 13 presented a two-dimensional projection of the trajectories in phase space for the first three main components, PC1, and PC3, for all complexes. Through their small occupancy in the phase space, as indicated by the PCA analysis along with additional molecular dynamics (MD) simulations, all the examined complexes demonstrated high stability according to these findings, which are consistent with the reported data from the 2D PCA representations.

### Free energy calculations

The binding free energies of the DPP4 selected compound and co-crystal are indicated in Table 3. According to the results, the binding free energies for selected ligand ZINC000003015356 was − 151.108 ± 11.170 kJ mol^−1^ was better than their related co-crystal ligand. As can be seen in Table 3, for ZINC000003015356, a large contribution of the total free energy (ΔG_binding_) was Van der Waals energy, and the electrostatic energy had a small contribution. These results proved that the binding site of DPP4 is mainly non-polar, which is consistent with the docking results. The solvent-accessible surface area (SASA) indicated the degree of solvent exposure^40^ that is reported in Table 3. The results of total binding energy for ZINC000003015356 proved that this compound is a suitable candidate as a DPP4 inhibitor.

**Table 3:** Binding free energy (KJ mol^−1^) for the selected compound of DPP4 and its related co-crystal.

Compounds	ΔE_vdw_	ΔE_ele_	ΔSASA^a^/nm^2^	ΔG_binding_
ZINC000003015356	− 194.242 ± 10.080	− 2.99 ± 2.76	− 17.833 ± 0.77	− 151.108 ± 11.170
N7F	− 166.415 ± 8.429	− 6.112 ± 3.633	− 17.071 ± 0.68	− 114.874 ± 11.984

### Pharmacokinetic profile

Recently researchers used some techniques to predict the drug ability of hit compounds such as the Lipinski rule of 5 (Ro5) and the ADME/T parameters^41^. Three parameters of Ro5 including molecular weight, hydrogen bond acceptor, and hydrogen bond donor, are related to the interaction of the ligands and the active site of the protein. The lipophilicity parameter (LogP) can be calculated experimentally unlike the other three parameters which are not related to the biophysical features of the target. Topological polar surface area (TPSA) indicates the ability of a compound to penetrate cells^42^. The ability of compounds to penetrate the cell membrane is reduced when the TPSA value is higher than 140 (Å^2^)^43^.

By using Swiss ADME and PreADMET software, the physicochemical and pharmacokinetics features of selected ligands and some of the FDA-approved DPP4 inhibitors were investigated. The amount of molecular weight, LogP, hydrogen bond donor, hydrogen bond acceptor, number of rotatable bonds, and topological polar surface area for ligands ZINC000003015356, ZINC000002876364, and ZINC000003007727 were in an acceptable range (Table 4).

**Table 4:** Physicochemical properties for selected DPP4 ligands, their co-crystals, and some FDA-approved drugs.

Compounds	MW (g mol^−1^)	LogP	HBD	HBA	TPSA (Å^2^)	n-RB	Lipinski violation
ZINC000003015356	447.48	1.22	1	5	89.87	8	0
ZINC000002876364	431.48	1.34	0	4	71.85	6	0
ZINC000003007727	431.48	1.34	0	4	71.85	6	0
N7F	489.57	2.39	1	5	105.76	5	0
Sitagliptin	407.31	2.52	1	10	77.04	6	0
Linagliptin	472.54	1.80	1	6	116.86	4	0
Saxagliptin	315.41	1.21	2	4	90.35	3	0
Vildagliptin	303.40	0.97	2	4	76.36	4	0
Rule of Lipinski	≤ 500	≤ 5	≤ 5	≤ 10	≤ 140	≤ 10	≤ 1

Predicting the interaction between drugs and the human body is shown by parameters such as HIA%, in vitro plasma protein binding%, and blood–brain barrier (BBB%) parameters that are related to the distribution of the drug in the body.

The HIA% value (91.35–97.94%) of selected ligands indicated the appropriate intestinal absorption. Besides, these ligands were desired to permeate the cell membrane, and the in vitro Caco-2 cell permeability for them was in an acceptable range. The negative values for in vitro skin permeability indicated that these compounds had no skin diffusion. Based on the results ZINC000003015356 showed a high affinity for binding to the plasma protein. The blood–brain barrier (BBB%) values of all compounds were in a proper range (0.02–0.19), therefore the compounds were not neurotoxic (Table 5)^44^.

**Table 5:** In silico ADME for selected DPP4 ligands, their co-crystals, and some of the FDA-approved drugs.

Entry	Absorption		Distribution		
compounds	%HIA	In vitro Caco-2 cell permeability (nm s^−1^)	In vitro skin permeability (lookup, cm h^−1^)	% in vitro plasma protein binding	%BBB
ZINC000003015356	96.72	29.62	− 4.01	91.52	0.03
ZINC000002876364	97.94	37.52	− 4.08	66.93	0.06
ZINC000003007727	97.59	41.06	− 3.83	92.82	0.19
N7F	97.22	25.52	− 3.11	79.97	0.26
Sitagliptin	97.05	21.68	− 3.07	54.32	0.03
Linagliptin	98.74	22.04	− 3.40	66.80	0.16
Saxagliptin	89.38	18.55	− 5.33	26.90	0.37
Vildagliptin	89.81	19.84	− 5.32	17.65	0.03

The obtained results for the toxicity profile of the selected ligands and some current DPP4 inhibitors are given in Table 6. The toxicity profiling for all studied compounds showed low toxicity against algae_. The Ames test values for all compounds indicated that these compounds were mutagens. The outputs of the carcino_mouse test exhibited that the studied ligands were not carcinogenic. Since blocking the hERG K + channels caused QT interval prolongation, subsequent sudden death may happen. Thus, this parameter was considered which showed medium risk for the studied compounds. In general, ZINC000003015356, in comparison with FDA-approved drugs, had no significant toxicity, therefore can be used as a proper candidate for inhibition of DPP4 enzyme.

**Table 6:** Toxicity profile for selected DPP4 ligands, their co-crystals, and some FDA-approved drugs.

Entry	Algae_at	Ames_test	Carcino_Mouse	hERG_inhibition
ZINC000003015356	0.02	Mutagen	Negative	Medium-risk
ZINC000002876364	0.04	Mutagen	Negative	Medium-risk
ZINC000003007727	0.04	Mutagen	Negative	Medium-risk
N7F	0.01	Mutagen	Negative	High-risk
Sitagliptin	0.03	Mutagen	Negative	Medium-risk
Linagliptin	0.01	Non-mutagen	Positive	High-risk
Saxagliptin	0.14	Mutagen	Negative	Low-risk
Vildagliptin	0.11	Mutagen	Negative	Low-risk

### DFT analysis

**Figure 14:**
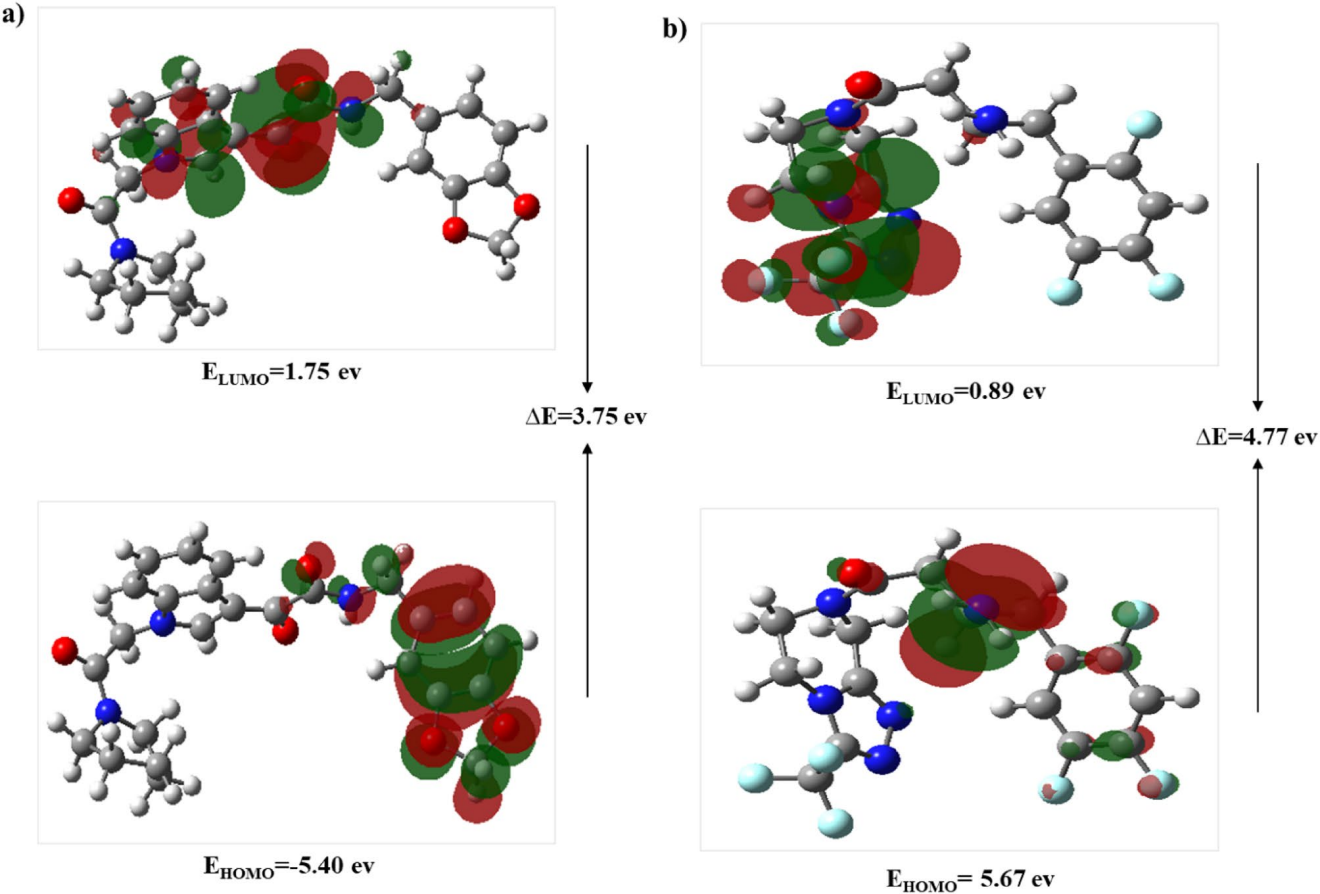
DFT calculated LUMO, HOMO, and their energies for (**a**) ZINC000003015356 and (**b**) Sitagliptin at the B3LYP/6–31 + G (d,p) level of theory.

The molecular orbitals, HOMO and LUMO, predicted reactivity and the physical, structural properties of compounds. The HOMO, LUMO energies and, energy gap between HOMO and LUMO are depicted in Fig. 14. The gap energies of Sitagliptin and ZINC000003015356 were obtained at 3.75 and 4.77 eV respectively. The results showed that the selective DPP4 inhibitor had higher chemical reactivity than Sitagliptin due to the lower energy gap between HOMO and LUMO.

**Figure 15:**
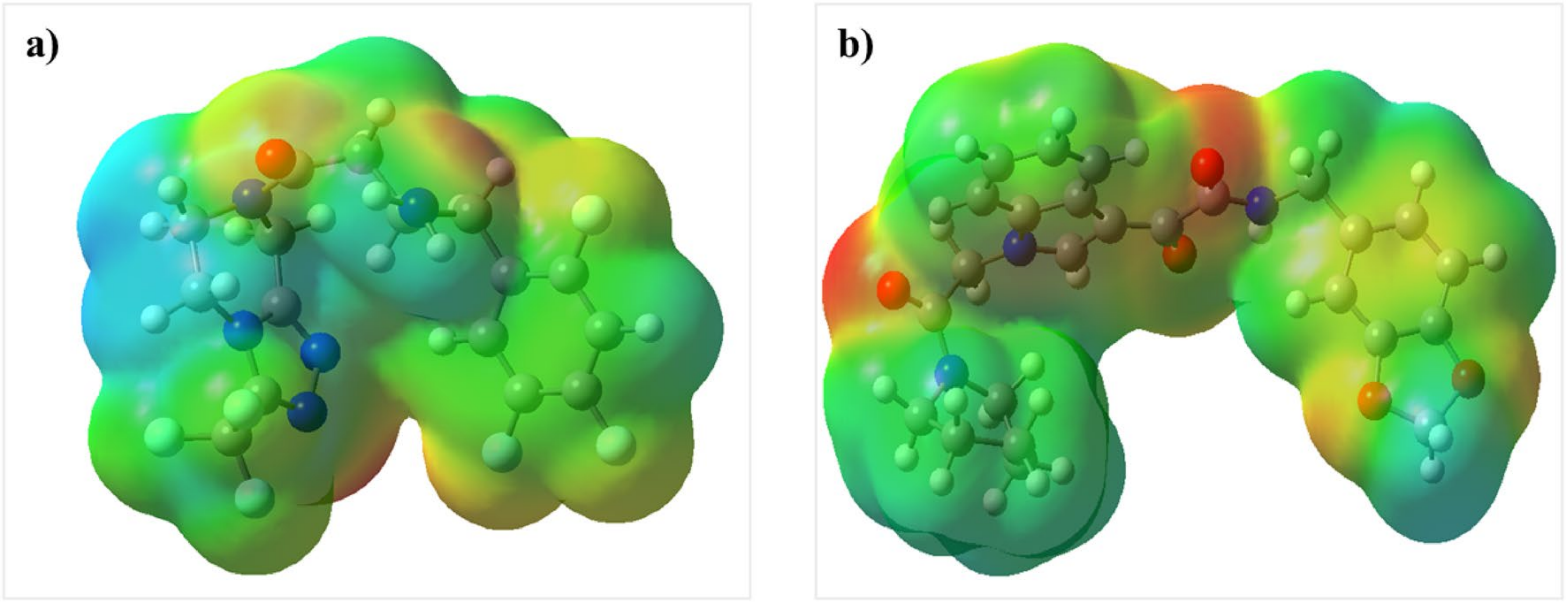
Geometry optimized as well as ESP of (**a**) ZINC000003015356 and (**b**) Sitagliptin at B3LYP/6–31 + G (d,p) level of theory.

The charge distribution of Sitagliptin and ZINC000003015356 is shown by electrostatic surface potential (ESP) energy (Fig. 15). The red spheres on the ESP graph represent the negative charge sites. The ESP map specified the balanced charge distribution in the ZINC000003015356 which facilitates the binding of the compound to biological enzymes. The global parameters are calculated at the B3LYP level and are shown in Table 7. The Hardness, softness, and electron affinity of ZINC000003015356 were obtained by using HOMO and LUMO energies. The values of entropy, enthalpy, and Gibbs energies showed more stability of Sitagliptin which was consistent with calculated HOMO, and LUMO energy values and energy gap.

**Table 7:** The calculated total energy (E_tot_), Enthalpy (H), Gibbs free energy (G), hardness (ɳ), softness (σ, and electron affinity (A) of ZINC000003015356 and Sitagliptin at B3LYP/6–31 + G(d,p) level of theory [^a^in Hartree/particle, ^b^in cal/mol K, ^c^in ev, ^d^in ev^−1^].

Entry	E_tot_^a^	H^a^	G^a^	S^b^	ɳ^c^	σ^d^	A^c^
ZINC000003015356	− 1499.321	− 949.320	− 1499.413	139.9	2.560	0.391	1.75
Sitagliptin	− 1557.82	− 1557.82	− 1557.90	172.59	2.38	0.42	0.89

## Conclusion

This study aimed to find a new high-affinity inhibitor for DPP4 by using computational methods. Using the ZINC database, target compounds were screened on ≥ 50 similarities to FDA-approved drugs. Thus, by performing molecular docking, compound ZINC000003015356 with the highest docking score and appropriate interaction with the active site of DPP4 was selected. Also, the molecular docking studies of ZINC000003015356 on DPP8 and DPP9 receptors indicated that this compound had no interaction with these proteins. Whereas, the docking results for FDA approved drugs on DPP8 and DPP9 enzymes represented that these compounds are placed in the binding site of DPP8 and have no interaction with DPP9. The molecular dynamics simulation and MM/PBSA calculations were done for a selected compound that confirmed the results of the molecular docking. According to simulation analysis, the key residues of Tyr662, Tyr666, Tyr547, and Trp629 were concluded necessary for a ligand to be a potent inhibitor of the DPP4 enzyme, subsequently, the results of pharmacokinetic properties and ADMET profile showed that the selected compound was a suitable candidate for drug likenesses. DFT analysis showed the high reactivity of the proposed ligand. To conclude, ZINC000003015356 can be considered a promising and selective DPP4 inhibitor candidate.

## Data Availability

The data sets used and analyzed during the current study are available from the corresponding author upon reasonable request. We have presented all data in the form of Tables and Figures.
